# Investigating the role of acute and repeated stress on remote ischemic preconditioning-induced cardioprotection

**DOI:** 10.22038/IJBMS.2019.36416.8678

**Published:** 2020-01

**Authors:** Sakshi Tyagi, Simranjot Kaur, Nirmal Singh, Amteshwar Singh Jaggi

**Affiliations:** 1 Department of Pharmaceutical Sciences and Drug Research Punjabi University, Patiala, India

**Keywords:** Acute stress, Adaptation, Cardioprotection, Cold-water immersion, Remote ischemic - preconditioning

## Abstract

**Objective(s):**

To study the effect of acute and repeated stress on cardioprotection-induced by remote ischemic preconditioning (RIPC).

**Materials and Methods:**

RIPC was induced by giving 4 short cycles of ischemia and reperfusion, each consisting of five min. The Langendorff’s apparatus was used to perfuse the isolated rat hearts by subjecting the hearts to global ischemia of 30 min and reperfusion of 120 min. The coronary effluent was collected to measure the levels of lactate dehydrogenase (LDH) and creatine kinase (CK) for the assessment of injury to the myocardium. Myocardial infarct size was measured by the use of triphenyl tetrazolium chloride. Acute stress was induced by subjecting the animals to cold immersion stress for 5 min. However, in the case of stress adaptation, rats were exposed to a homotypic stressor (cold-water immersion stress) each of 5 min duration for five consecutive days.

**Results:**

RIPC demonstrated a significant decrease in ischemia-reperfusion-induced myocardial injury in terms of decrease in LDH, CK, and infarct size. However, acute stress for five minutes prior to RIPC significantly abolished its cardioprotective effects. Exogenous administration of adenosine restored RIPC-induced cardioprotective effects in the presence of acute stress. On repeated stress exposure for 5 days, stress adaptation was noted, and there was no effect of repeated stress exposure on RIPC-induced cardioprotection. However, the cardioprotective effects of adenosine were absent in the case of rats subjected to repeated episodes of stress.

**Conclusion:**

Acute stress, but not repeated stress exposure, may alter the release of adenosine during RIPC, which may be manifested in the form of reduced cardioprotection during ischemic-reperfusion injury.

## Introduction

The restoration of blood flow in the form of reperfusion following prolonged ischemia exacerbates the injury, which is termed as reperfusion injury. Ischemia, along with reperfusion injury, is responsible for the high incidence of mortality in myocardial infarction and ischemic stroke patients (1). Various strategies have been devised by the scientists in order to minimize the degree of myocardial injury induced in response to ischemia-reperfusion, including ischemic conditioning induced by short-term ischemia-reperfusion episodes (conditioning stimulus), which protects the heart from the injury due to sustained ischemia-reperfusion (2, 3). Based on the delivery time and the site where the conditioning stimulus is applied, ischemic conditioning has been categorized into following types: ischemic preconditioning (heart is subjected to conditioning stimulus prior to the onset of sustained ischemic insult) (2), ischemic postconditioning (heart is subjected to conditioning stimulus at the onset of reperfusion), remote ischemic preconditioning (non-cardiac tissue is subjected to conditioning stimuli prior to sustained ischemia) (4-6), remote ischemic preconditioning (non-cardiac tissue is subjected to conditioning stimuli during the time of cardiac ischemia), and remote ischemic postconditioning (non-cardiac tissue is subjected conditioning stimuli at the onset of reperfusion) (7-9). Apart from the short-term cycles of ischemia and reperfusion, administration of pharmacological agents including adenosine, nitric oxide donors, thrombin, bradykinin, opioids, statins, certain volatile anesthetics, and K_ATP_ channel openers is also shown to impart cardioprotection against ischemic-reperfusion damage for inducing pharmacological preconditioning (10, 11).

Amongst different types of ischemic conditioning, remote ischemic preconditioning (RIPC) has received considerable attention, and its cardioprotective effects have been documented in patients undergoing heart surgery (12). However, the cardioprotective effects of RIPC have not been consistently observed in the patients in all conditions. Indeed, the cardioprotective effects are influenced by different factors, including age (13); comorbidities including stable angina, type II diabetes, cardiac hypertrophy (14, 15); pharmacological agents including oral hypoglycemic agents (16) and anesthetics (17).

Stress is a state of threatened homeostasis, and it has been shown to induce a number of neuropathological, cardiovascular, and metabolic disorders (18). It is possible that like aging, diabetes and other factors, and exposure to stress may also affect the outcome of remote preconditioning-induced cardioprotection. However, there is no such report documenting the role of acute or repeated stress on remote preconditioning-induced cardioprotection. Adenosine is a product of the metabolism of adenosine triphosphate, with several essential physiological roles (19, 20). Furthermore, adenosine is a crucial mediator of RIPC, and its release during RIPC is associated with increased development of cardiac resistance towards sustained ischemia-reperfusion-induced injury (21, 22). Therefore, this study was designed to **(a) **explore the effect of acute and repeated stress exposure on RIPC-induced cardioprotection and **(b)** delineate the effect of adenosine in the modulation of RIPC due to stress in rats. 

## Materials and Methods

***Animals, drugs, and chemicals***

In the present study, Wistar rats (Lala Lajpat Rai University of Veterinary and Animal Sciences, Haryana, India) weighing 180–250 g of either sex were used. Animals were fed standard laboratory diet (Ashirwad Industries, Kharar, Mohali, Punjab) and water *ad libitum*. They were housed in the departmental animal house and were exposed to natural cycles of light and dark. The experimental protocol was approved by the Institutional Animal Ethics Committee (approval no. 107/GO/ReBi/S/99/CPCSEA/2018-50), and experiments were carried out as per the guidelines of the Committee for the Purpose of Control and Supervision of Experiments on Animals (CPCSEA), Government of India. Adenosine (Samarth Life Sciences Pvt. Ltd., India) was used in the present study and administered through the IP route after dissolving it in water. The dose of adenosine employed in the present study was obtained from our previous study (23).

***Remote ischemic preconditioning***

The animals were anesthetized by the administration of thiopental sodium (50 mg/kg) through the IP route. Ischemia to the limb was delivered by tying the pressure cuff on the hind limb of the rat, and the cuff was inflated for 5 min with air up to 150 mm of Hg. Thereafter, the cuff was deflated to release the pressure and institute reperfusion. Four such episodes of inflation (ischemia) and deflation (reperfusion), each comprising 5 min, were employed to induce remote ischemic preconditioning (9, 24).

***Induction of cold-water immersion stress***

Stress was induced in rats by placing them individually in a tank of water, at the temperature of 15±2 ^°^C, for 5 min. During this period, the rat remained in an upright position, keeping its head above the water level. The time of 5 min was selected because after this period of time, animals started to sink (25). The influence of stress on rats was assessed by measuring locomotor activity in an actophotometer test for five minutes after 25 min of stress exposure.

***Isolated rat heart preparation***

Heparinization (500 IU/kg, IP) was done about 20 min before killing the rat by cervical dislocation under anesthesia, and the heart was excised and rapidly mounted on the Langendorff’s apparatus. The isolated rat heart was perfused in a retrograde manner at a constant pressure of 70 mm Hg with Krebs-Henseleit solution, maintained at pH 7.4, optimum temperature of 37 ^°^C with 95% O_2_, and 5% CO_2_. The flow rate varied between 7 and 8 ml/min. 30 min of global ischemia was induced by blocking the inflow of the Krebs-Henseleit solution. Afterward, 120 min of reperfusion was performed in which the flow of the Krebs-Henseleit solution was reinstituted. The coronary effluent was collected before ischemia and during different time intervals of reperfusion, i.e., at the start of reperfusion, 5 min after reperfusion, and 30 min after reperfusion for the biochemical estimations of LDH and CK (6, 23).

***Infarct size estimation***

The heart was removed from the Langendorff apparatus after reperfusion and was kept overnight in the freezer. Thereafter, the frozen heart was sliced into uniform sections, each of 2–3 mm thickness and slices were stained with 1% TTC (triphenyl tetrazolium chloride). The infarcted (unstained or yellow) and non-infarcted (stained red) portions were identified, and infarct size was expressed in % volume and % weight (26-29). 

***Assessment of lactate dehydrogenase (LDH)***

In the samples of coronary effluent, the levels of lactate dehydrogenase were quantified by a 2,4-dinitrophenylhydrazine method. The coronary samples for LDH estimation were collected at various intervals, i.e., before ischemia, immediately after reperfusion and 30 min after reperfusion (30).

***Assessment of creatine kinase (CK)***

In the samples of coronary effluent, the levels of creatine kinase (CK) were quantified using an ELISA kit for the estimation of CK levels (Agappe Diagnostics Ltd. Kerala, India). The coronary effluent samples were collected at various intervals, i.e. before ischemia and 5 min after reperfusion.

***Experimental protocol***

Seven groups (n=6) were employed:

*Group I (Control)*

Animals were administered water for injection 40 min before isolation of the heart from rats. Thereafter, the heart was mounted on the Langendorff’s apparatus and stabilized for 10 min. Afterward, the isolated hearts were subjected to global ischemia of 30 min and reperfusion for 120 min.

*Group II (RIPC)*

Thiopental sodium (45 mg/kg, IP) was used to anesthetize the animals, and a neonatal pressure cuff was tied around the hind limb of the rat to induce RIPC as described in section 2.2. The time duration to induce RIPC was 40 min, and after completion of RIPC protocol, the heart was excised and subjected to ischemia and reperfusion as described for group I.

*Group III (Acute stress+RIPC)*

The rat was placed in a water tank for five minutes to induce cold-water immersion stress (acute stress), and thereafter, the rat was subjected to RIPC for 40 min. Afterward, the heart was removed and subjected to ischemia and reperfusion as described for group I.

*Group IV (Acute stress+Adenosine+RIPC)*

Rats were subjected to acute stress, and adenosine (4 mg/kg, IP) was administered. After 30 min of adenosine dosing, rats were subjected to RIPC. Afterward, the heart was subjected to ischemia and reperfusion, as described for group I.

*Group V (Adenosine+RIPC)*

Adenosine was administered to non-stress-subjected rats, and after 30 min of adenosine dosing, the rat was subjected to RIPC. Afterward, the heart was subjected to ischemia and reperfusion, as described for group I.

*Group VI (Repeated stress+RIPC)*

Rats were subjected to repeated episodes of cold-water immersion stress for 5 min each for five consecutive days. On the 5^th^ day, RIPC was performed, and the rat heart was isolated. Thereafter, the heart was subjected to 30 min of global ischemia and 120 min of reperfusion as described in group I.

*Group VII (Repeated stress+Adenosine+RIPC)*

Cold-water immersion stress was induced in rats for five days. On the 5^th^ day, after the last episode of stress, adenosine (4 mg/kg, IP) was administered. After 30 min of adenosine dosing, the rat was subjected to RIPC. Thereafter, the heart was isolated and subjected to ischemia and reperfusion as described for group I.

***Statistical analysis***

The results were expressed as mean±standard deviation (SD). One-way ANOVA for infarct size, Two-way ANOVA for LDH, and CK followed by Bonferonni’s *post hoc *test were performed. Bonferonni’s test is one of the more commonly employed multiple comparison *post hoc *tests following ANOVA, which is used to identify the statistically significant differences among different groups. The outcome value of *P<*0.05 was considered statistically significant.

## Results

***Influence of different interventions on myocardial infarct size***

**Figure 1:**
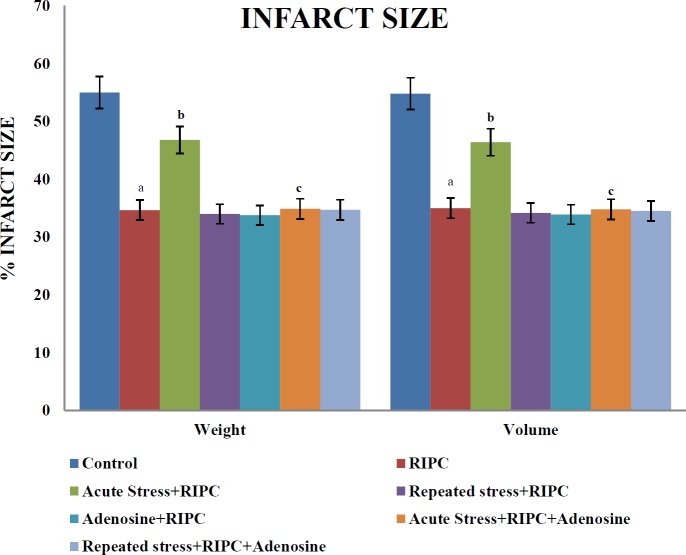
The effect of different pharmacological interventions on myocardial infarct size

In isolated rat hearts, the myocardial injury was measured in terms of significant increase in infarct size in response to ischemia of 30 min and reperfusion of 120 min. However, brief episodes of ischemia and reperfusion in the form of RIPC resulted in significant attenuation of ischemia-reperfusion injury-induced increase in myocardial infarct size. A single episode of cold immersion stress (acute stress) prior to RIPC significantly abolished its infarct sparing effects. Administration of adenosine (4 mg/kg, IP) following acute stress exposure restored the infarct size attenuating effects of RIPC. Repeated exposures to the same stressor for five days did not affect the reduction in infarct size induced by RIPC. Furthermore, administration of adenosine did not show any significant effect on RIPC-induced decrease in infarct size in repeated stress or non-stress-subjected rats (Figure 1).

***Influence of different interventions on LDH release in coronary effluent***

**Figure 2:**
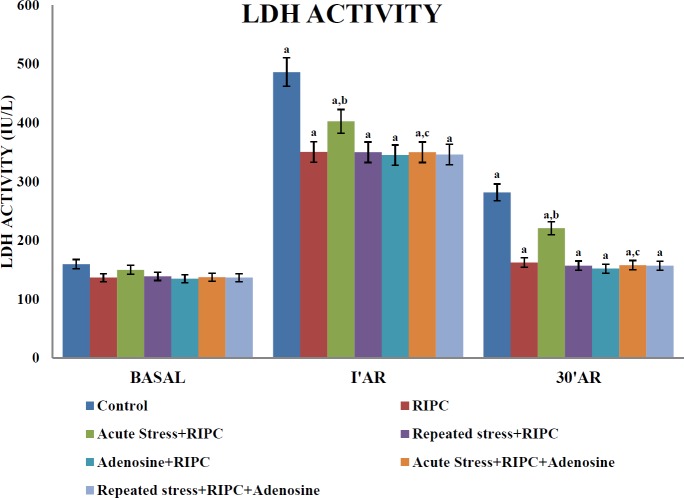
The effect of different pharmacological interventions on lactate dehydrogenase activity (LDH).

In the coronary effluent, the release of LDH was increased by 30 min of global ischemia and 120 min of reperfusion. However, LDH release was significantly reduced at different periods of time in RIPC-subjected rats. A single episode of cold immersion stress significantly abrogated the effects of RIPC on LDH release. Administration of adenosine (4 mg/kg, IP) restored the RIPC effects including attenuation of LDH release in acute stress-subjected rats. However, repeated stress did not show any significant effect on RIPC-induced decrease in LDH release. Moreover, administration of adenosine did not show any change in the effects of RIPC on LDH release in repeated stress-subjected or non-stress-subjected rats (Figure 2). 

***Influence of different interventions on the release of CK***

**Figure 3:**
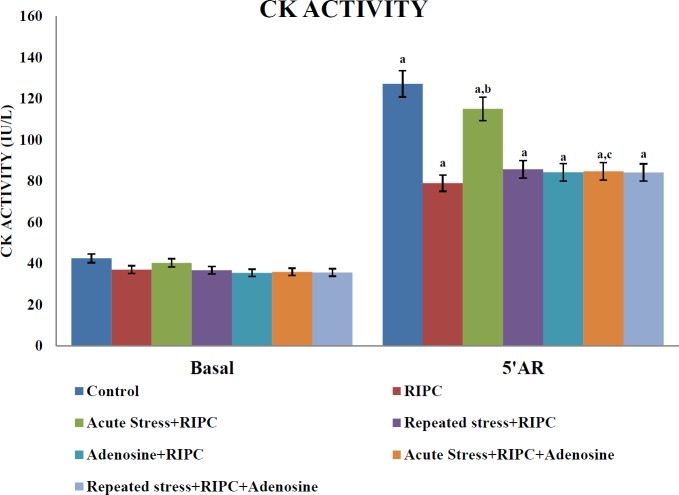
The effect of different pharmacological interventions on creatine kinase (CK) activity

Global ischemia of 30 min and reperfusion for 120 min resulted in a significant increase in the release of CK in the samples of the coronary effluent measured at different time intervals. RIPC significantly attenuated ischemia-reperfusion-induced elevation in levels of CK release in the samples of the coronary effluent in comparison to the control group. RIPC-induced attenuation of CK release was significantly decreased by a single episode of stress. Moreover, adenosine (4 mg/kg) administration through the IP route following acute stress exposure restored the attenuating effects of RIPC on CK release. However, repeated episodes of cold-water immersion stress for five days did not show any effect on RIPC-induced attenuation of CK release. Administration of adenosine did not show any changes in the effects of RIPC on CK release in repeated stress or non-stress-subjected rats (Figure 3).

***Modulation of locomotor activity due to cold-water immersion stress***

**Figure 4:**
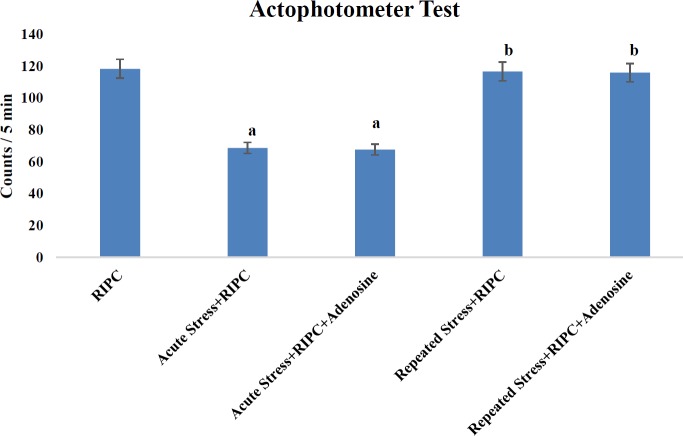
Assessment of the locomotor activity in terms of counts in 5 min time interval in the actophotometer test for evaluating the stress adaptive effect of cold-water immersion stress. The locomotor activity was assessed immediately prior to RIPC. Values are expressed as mean±SD with n= 6 in each group. The data were analyzed by One Way ANOVA followed by Post hoc analysis using Tukey’s multiple comparisons test, F (4, 10)= 210.1; *P*<0.05 for evaluating the effect on locomotor activity; a= *P*<0.05 vs RIPC; b= *P*<0.05 vs Acute stress + RIPC

Rats subjected to a single episode of cold-water immersion stress for 5 min resulted in a significant decrease in the locomotor activity on day 1. However, repeated exposure to homotypic stressor for 5 consecutive days (repeated stress) led to restoration of the locomotor activity, which was significantly higher in comparison to rats subjected to acute stress. Administration of adenosine did not produce any significant effect on the locomotor activity in acute stress, repeated stress, or non-stress-subjected rats (Figure 4).

## Discussion

In this investigation, global ischemia of 30 min and reperfusion of 120 min produced significant injury to the myocardial region, assessed in the form of elevation in the release of LDH and CK in the samples of the coronary effluent along with an increase in myocardial infarction. Myocardial injury was detected using well documented biochemical markers LDH and CK, as their release reliably indicates the extent of heart injury. An increase in the release of biochemical markers in response to ischemia-reperfusion in association with myocardial infarction is in concordance with the studies earlier reported by our laboratory (31, 32). Remote preconditioning in the form of short episodes of ischemia-reperfusion to the hind limb significantly produced myocardial protection in terms of decrease in LDH, CK release, and myocardial infarct size in ischemia-reperfusion subjected rats. The present study results showing protective effects of remote preconditioning are supported by earlier published studies from our laboratory (29, 32). In our previous investigations, animals of both sexes were used, and there was no significant effect of gender on the outcome of remote preconditioning (31-33). Accordingly, in this study animals of both sexes were used.

To investigate the effects of stress exposure on remote preconditioning, rats were subjected to single or repeated episodes of cold-water immersion stress prior to subjecting to remote preconditioning. Cold-water immersion stress is one of the most commonly used animal models to study the various aspects related to stress (34). Various time intervals, such as 3 min, 5–7 min, and 15 min, have been reported by different research groups for the induction of acute stress in cold-water immersion model (34). In this present study for induction of acute stress, the rats were subjected to a single episode of cold-water immersion for five minutes because the animals were starting to sink after this period of time, which is consistent with our earlier report (25). In the present study, a single episode of stress to rats significantly abolished RIPC-induced protective effects on the heart. Studies have shown that there are many factors that may affect the positive effects of remote ischemic preconditioning, including age (13), stable angina, type II diabetes (14), cardiac hypertrophy (15), oral hypoglycemic agents (16, 35), and anesthetics (36). However, it is the first report suggesting the attenuation of remote preconditioning-induced cardioprotective effects in response to acute stress exposure. 

To explore the mechanisms that may participate in attenuating the cardioprotective effects of RIPC during acute stress exposure, the role of adenosine was investigated. In the present study, exogenous administration of adenosine in acute stress-subjected rats restored the cardioprotective effects of RIPC. Adenosine is an extracellular purine nucleoside, which is implicated in a wide variety of functions in relation to physiological systems such as vasodilation, diminishing blood pressure, and heart rate. Adenosine receptors are present on various body parts including the heart, and their roles in different types of preconditioning including RIPC, have been reported (6). It has been observed that during short episodes of ischemia of RIPC, adenosine is released which acts on the adenosine receptors of the heart to produce cardioprotection (23). The results of this present study showing the restoration of RIPC-induced cardioprotective effects during acute stress in the presence of adenosine suggest that there may be impairment in adenosine release during RIPC in response to acute stress. It may also be stated that acute stress may alter the release of adenosine during RIPC, which may be manifested in the form of reduced cardioprotection during ischemia-reperfusion injury.

In contrast to acute stress exposure, repeated exposure to homotypic stressor for five consecutive days did not alter RIPC-induced cardioprotective effects. This suggests that repeated exposure to stress may not abolish the cardioprotective effects of RIPC. It is well documented from studies of our laboratory and others that repeated exposure to stress may be associated with the development of stress adaptation (25, 37). Indeed, repeated stress-subjected animals may not respond to stress stimuli as naïve animals do. There is a reduction in stress sensitivity in these animals, and there is normalization of acute stress-induced alterations in the body. The present study result also shows the normalization of locomotor activity in an actophotometer test (a behavioral parameter to assess stress response) on the 5^th^ day before instituting RIPC in comparison to acute stress-subjected rats. The normalization of locomotor activity indicates that stress adaptation is induced, which is in consonance with our previous studies (25, 37). Therefore, it is hypothesized that a single episode of acute stress-induced alteration in RIPC-induced cardioprotective effects may not be noticed in repeated stress-subjected animals due to stress adaptation. Furthermore, administration of adenosine in rats subjected to repeated episodes of stress did not modulate RIPC-induced cardioprotection. This suggests that unlike acute stress subjected rats, there may not be alteration in adenosine release in repeated stress subjected rats. 

It is also worth mentioning that adenosine did not enhance RIPC-induced cardioprotection, which possibly suggests that RIPC may have produced optimal cardioprotection, and further enhancement in cardioprotection is not possible with concomitant administration of another cardioprotective agent. It also suggests that RIPC and pharmacological preconditioning with adenosine may involve same mechanisms, i.e., cardioprotective effects of RIPC involves release of adenosine. Therefore, further exogenous administration of adenosine may not be able to enhance the optimal cardioprotection due to endogenous release of adenosine during RIPC. The major limitation of this study is that the changes in the adenosine levels in the plasma were not monitored, which could have further supported the conclusions of the study. Nevertheless, the major highlight of this study is the finding that acute stress can attenuate the cardioprotective effects offered by RIPC, and institution of appropriate pharmacological agents such as adenosine may restore the attenuated cardioprotective effects of RIPC in stressful conditions. 

The data were statistically analyzed by using One-way ANOVA followed by Tukey’s multiple range tests. Values were expressed as mean±SD with n=6 in each group. For volume method, F (6, 35)=60.94; for weight method, F (6, 35)= 62.7; a= *P*<0.05 vs control; b= *P*<0.05 vs RIPC; c= *P*<0.05 vs Acute Stress + RIPC. I´AR stands for ‘immediately after reperfusion’ and 30´AR for ‘30 min after reperfusion’

The data were statistically analyzed by using Two-way ANOVA, followed by the Bonferroni test. Values were expressed as mean ± SD with n=6 in each group. RIPC- Remote ischemic preconditioning; for treatment, F (6, 105) = 71.74; for time, F (2, 105) = 1091; a= *P*<0.05 vs basal; b= *P*<0.05 vs RIPC; c = *P*<0.05 vs Acute Stress + RIPC. triphenyl tetrazolium stands for ‘immediately after reperfusion’ and 30´AR for ‘30 min after reperfusion’

The data were statistically analyzed by using Two-way ANOVA, followed by the Bonferroni test. Values were expressed as mean±SD with n=6 in each group. For treatment, F (6, 70)= 56.56; for time, F (1, 70) = 3043; a= *P*<0.05 vs basal; b= *P*<0.05 vs RIPC; c= *P*<0.05 vs Acute Stress+RIPC. I´AR stands for ‘immediately after reperfusion’ and 30´AR for ‘30 min after reperfusion’

## Conclusion

Acute stress may abolish RIPC-induced cardioprotective effects, and adenosine may restore the attenuated cardioprotective effects of RIPC suggesting that acute stress may impair the release of adenosine during remote preconditioning. On the other hand, repeated stress may not influence remote preconditioning-induced cardioprotection, possibly due to development of stress adaptation to repeated stress exposure.
